# A Case of Prenatally Diagnosed Uhl’s Anomaly with Absent Pulmonary Valve Leaflets and Dysplastic Tricuspid Valve

**DOI:** 10.3390/children8030190

**Published:** 2021-03-03

**Authors:** Taehong Kim, Hoon Ko, Joung-Hee Byun, Hyoung Doo Lee, Hyungtae Kim, Kwangho Choi, Joo-Young Na

**Affiliations:** 1 Department of Pediatrics, Pusan National University Yangsan Hospital, Yangsan-si 50612, Korea; md3728@pednet.co.kr (T.K.); peddrkh@gmail.com (H.K.); africa3217@naver.com (J.-H.B.); 2 Department of Thoracic and Cardiovascular Surgery, Pusan National University Yangsan Hospital, Yangsan-si 50612, Korea; 2719k@naver.com (H.K.); ppippo77@gmail.com (K.C.); 3 Department of Pathology, Pusan National University Yangsan Hospital, Yangsan-si 50612, Korea; pdrdream@gmail.com

**Keywords:** Uhl’s anomaly, fetal echocardiography, pulmonary valve leaflets

## Abstract

Uhl’s anomaly is a very rare malformation of unknown cause, characterized by complete or partial absence of the right ventricular myocardium. The cardiac malformation causes progressive right heart failure, increased right-sided cardiac pressure, massive peripheral edema, and ascites. Patients usually present in infancy and rarely survive to adulthood. Previously, diagnosis was made at post-mortem evaluation, but advances in cardiac imaging now permit diagnosis during fetal life. We report a case of Uhl’s anomaly in a newborn baby imaged at 23 + 3 weeks of gestation by fetal echocardiography. There was an aneurysmally dilated thin-walled right ventricle with hypertrophy of the right ventricular apical muscles, the tricuspid valve was dysplastic, and the pulmonary valve leaflets were absent.

## 1. Introduction

Uhl’s anomaly is a very rare cardiac malformation that is characterized by complete or partial absence of the right ventricular myocardium, which is replaced by a parchment-like endocardial tissue and epicardial tissue, with no evidence of a myocardium. It was described by Henry Uhl in 1952 after performing an autopsy on an 8-month-old infant [1,2].

Previously, most cases of Uhl’s anomaly have been found at post-mortem evaluation, but nowadays, the diagnosis can be made during life, including fetal life, due to the advancements in cardiac imaging equipment [3].

We report a case of Uhl’s anomaly with a dysplastic tricuspid valve and absent pulmonary valve leaflets imaged at 23 + 3 weeks of gestation using fetal echocardiography.

## 2. Case Report

**Figure 1:**
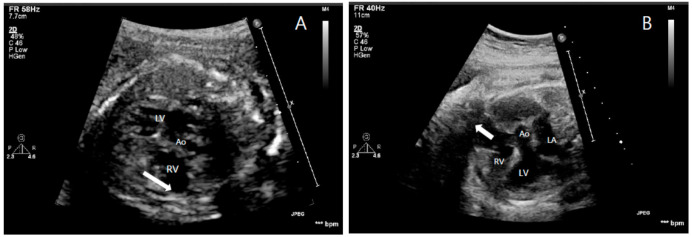
Fetal echocardiogram at 23 + 3 weeks of gestation (**A**) shows dysplastic RV cavity and focal thinning of RV free wall myocardium (white arrow) and at 35 + 1 weeks of gestation (**B**) shows aneurysmal dilatation of the RV (white arrow). RV, right ventricle; LV, left ventricle; LA, left atrium; Ao, aorta.

On 25 March 2020, a 31-year-old woman (gravida 1, para 0) was referred from another hospital at a gestational age of 23 + 3 weeks because of a suspicion of heart anomaly. No significant medical or family history was suggestive of any structural heart disease. A fetal echocardiogram performed at the gestation age of 23 + 3 weeks revealed dysplastic right ventricular cavity with focal thinning, and dilatation of the myocardium of the right ventricular free wall, with bulging of the interventricular septum into the left ventricular outflow tract (LVOT). The visceroatrial situs was solitus and the aortic arch was normal with no arch obstruction. The tricuspid valve was nearly atretic and had several small perforations with regurgitant flow. The main pulmonary artery (MPA) arose from the right ventricle (RV) normally and branched to both sides with good perfusion. The ductus arteriosus connected to the MPA with right to left shunt (Figure 1A).

At 35 + 1 weeks of gestation, on 15 July 2020, fetal echocardiography showed a dilated RV that had a dysplastic cavity, with focal thinning of the right ventricular free wall and the interventricular septum bulged into the LVOT. There were no pulmonary valve leaflets with free pulmonary regurgitation. Other findings were similar to those observed in previous fetal echocardiograms (Figure 1B).

On 29 July 2020, a male neonate was delivered by cesarean section. He weighed 3.14 kg (50~75 percentile), length was 46 cm (within the 25 percentile), and his Apgar scores at the first and fifth minutes were 3 and 5 points, respectively. Oxygen saturation in room air was below 80%. After birth, the neonate was severely dyspneic and was intubated immediately and placed on mechanical ventilation. 

**Figure 2:**
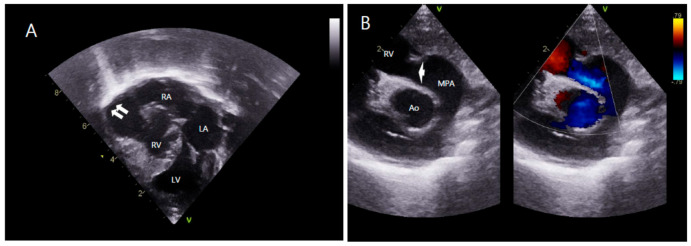
Transthoracic echocardiogram. (**A**) Apical four-chamber view shows aneurysmal dilatation of RV (white arrow) with apical muscular hypertrophy. (**B**) Parasternal long-axis view shows absence of pulmonary valve leaflets (white arrow) with free pulmonary regurgitation. LA, left atrium; LV, left ventricle; RA, right atrium; RV, right ventricle; Ao, aorta; MPA, main pulmonary artery.

Chest radiography showed cardiomegaly and a cardiothoracic ratio of 0.75. A transthoracic echocardiogram (Figure 2) showed that the situs was solitus and the entire RV except the apical portion was aneurysmally dilated. Apical hypertrophy of the myocardium of the RV was observed. The tricuspid valve was atretic with no movement. The site of the tricuspid valve had several small holes with continuous severe regurgitant flow. The pulmonary annulus was normal in size (7.1 mm, Z-score −0.27), but there were no pulmonary valve leaflets. MPA (11.9 mm, Z-score 2.75), right PA (7.78 mm, Z-score 3.39), and left PA (6.76 mm, Z-score 2.88) had good blood flow. The mitral annulus was 16.4 mm (Z-score 2.45). The aortic arch was left-sided and the annulus was 10.1 mm (Z-score 4.38). There was a large patent ductus arteriosus with a left to right shunt. 

**Figure 3:**
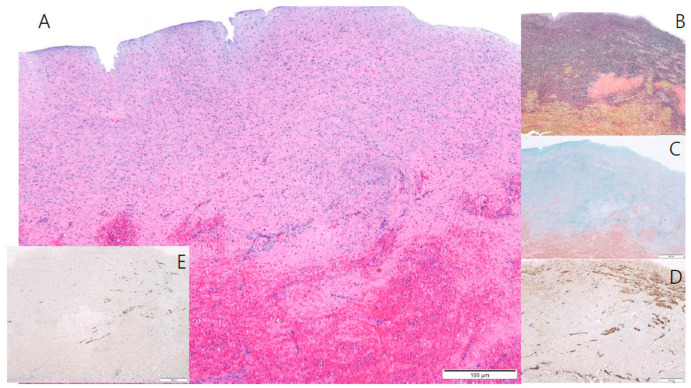
Microscopic examination of the RV free wall. (**A**) Microscopic findings of the RV reveal an elastic arterial tissue with hemorrhage (H & E stain, ×40). (**B**) The middle layer of the RV is composed of elastic fibers and multi-focal fragmentations and loss of elastic fibers (Verhoeff’s elastic stain, ×40). (**C**) The middle layer of the RV comprises large amounts of proteoglycans (Alcian blue stain, ×40). (**D**) Smooth muscle cells are seen multi-focally (Smooth muscle actin stain, ×40). (**E**) A few scattered cardiomyocytes are also seen (Desmin immunostain, ×40). RV, right ventricle.

On the eighth day of life, he underwent right modified Blalock-Taussig shunt with Gore-Tex tube graft, tricuspid valve and pulmonary valve obliteration, resection of the right ventricular aneurysmal free wall, and atrial septectomy. Histological examination of the right ventricle demonstrated the absence of the RV myocardium (Figure 3). During the 3-month follow-up, the patient was awaiting a staged single-ventricle palliation involving a bidirectional Glenn procedure. 

## 3. Discussion

Congenital hypoplasia of the right ventricular myocardium, also known as “parchment heart” or Uhl’s anomaly, is a rare congenital heart anomaly. It was first described as a thin, parchment-like heart where the ventricles are thin and dilated, in Osler’s Principles and Practice of Medicine in 1905 [4]. Later, in 1952, Uhl reported an autopsy of a newborn infant who died of RV failure [1]. In 1993, Gerlis et al. reported that many cases of arrhythmogenic right ventricular dysplasia were incorrectly classified as Uhl’s anomaly and discussed 84 real cases of Uhl’s anomaly [5]. 

The cause of the absence of the right ventricular myocardium is not fully known. It was originally attributed to an embryologic failure of right cardiogenic fold development, leading to a congenital absence of the RV myocardium [3,6]. Another hypothesis was the selective but uncontrolled apoptotic destruction of the RV myocardium during the perinatal period after complete cardiac development [3,7]. Some authors have suggested that overexpression of vascular endothelial growth factor, mainly by cardiomyocytes, may be responsible for impairment of ventricular myocardial development [2,8].

In Uhl’s anomaly, absence of the right ventricular myocardium leads to a lack of contraction and, consequently, the chamber acts as a connecting channel between the right atrium and the pulmonary artery. The pulmonary circulation is maintained by the pumping action of the right atrium, whereas the RV balloons aneurysmally during systole and the right atrium eventually enlarges [9].

Congestive heart failure and arrhythmia are the main modes of clinical features. Congestive heart failure is by far the most frequent symptom. It may be associated with severe peripheral edema, massive pleural effusion, or cardiac tamponade [5,10]. Arrhythmias and conduction disturbances are not a frequent presentation of Uhl’s anomaly, probably due to the absence of residual foci to initiate or transmit anomalous electrical activity [5]. The severity of the clinical manifestations of Uhl’s anomaly varies from premature death in childhood to rare reports of asymptomatic adult patients [11].

In the past, diagnosis was performed during autopsy. However, diagnosis is currently made frequently using imaging modalities, such as an echocardiography or cardiac magnetic resonance imaging, and can be confirmed by a myocardial biopsy [7,9,12]. The key features on echocardiography were thinning of the RV wall with marked dilatation of RV and significant reduction in RV contractility. Right atrial enlargement and hypertrophy is also observed, with a normal left chamber [6]. Cardiac magnetic resonance imaging reveals an extremely thin-walled RV with almost complete absence of the myocardium of the right ventricular free wall and a paucity of apical trabeculations with normal left ventricular myocardium. There is no fibro-fatty infiltration of the free wall [9]. The typical histological description in Uhl’s anomaly is a transparent right ventricular free wall due to apposition of the endocardium and the epicardium without any intervening myocardium, infiltration, or inflammation [1,2].

The prognosis of Uhl’s anomaly is poor but varies in relation to the extent of the destruction of the right ventricular myocardium and the heart conduction system. The treatment of Uhl’s anomaly is palliative with some controversy. There are several descriptions of various successful surgical repairs of Uhl’s anomaly [13]. The most frequent surgical managements are RV exclusion with closure of the tricuspid valve orifice, atrial septostomy, and bi-directional Glenn shunt [14]. The “one and a half” ventricular repair is a RV reductoplasty combined with atrial septostomy and bi-directional Glenn shunt [15]. Heart transplantation has also been reported [16]. However, there is no consensus on the definitive approach.

In conclusion, Uhl’s anomaly is an extremely rare condition. Even though this anomaly has been known for a long time, its etiology is still unknown. We report the case of a newborn infant who had Uhl’s anomaly with tricuspid valve dysplasia and absence of pulmonary valve leaflets. The patient was diagnosed using fetal echocardiography. A greater understanding and accurate diagnosis of Uhl’s anomaly during fetal life can help in the management of such neonates.

## Data Availability

The data presented in this study are available on request from the corresponding author. The data are not publicly available due to privacy restrictions.
